# Machine Learning–Based Prediction of Changes in the Clinical Condition of Patients With Complex Chronic Diseases: 2-Phase Pilot Prospective Single-Center Observational Study

**DOI:** 10.2196/52344

**Published:** 2024-04-19

**Authors:** Celia Alvarez-Romero, Alejandro Polo-Molina, Eugenio Francisco Sánchez-Úbeda, Carlos Jimenez-De-Juan, Maria Pastora Cuadri-Benitez, Jose Antonio Rivas-Gonzalez, Jose Portela, Rafael Palacios, Carlos Rodriguez-Morcillo, Antonio Muñoz, Carlos Luis Parra-Calderon, Maria Dolores Nieto-Martin, Manuel Ollero-Baturone, Carlos Hernández-Quiles

**Affiliations:** 1 Computational Health Informatics Group, Institute of Biomedicine of Seville, Virgen del Rocío University Hospital, Consejo Superior de Investigaciones Científicas, University of Seville Spain; 2 Institute for Research in Technology (IIT) ICAI School of Engineering Comillas Pontifical University Madrid Spain; 3 Internal Medicine Department Virgen del Rocio University Hospital Sevilla Spain

**Keywords:** patients with complex chronic diseases, functional impairment, Barthel Index, artificial intelligence, machine learning, prediction model, pilot study, chronic patients, chronic, development study, prognostic, diagnostic, therapeutic, wearable, wearables, wearable activity tracker, mobility device, device, physical activity, caregiver

## Abstract

**Background:**

Functional impairment is one of the most decisive prognostic factors in patients with complex chronic diseases. A more significant functional impairment indicates that the disease is progressing, which requires implementing diagnostic and therapeutic actions that stop the exacerbation of the disease.

**Objective:**

This study aimed to predict alterations in the clinical condition of patients with complex chronic diseases by predicting the Barthel Index (BI), to assess their clinical and functional status using an artificial intelligence model and data collected through an internet of things mobility device.

**Methods:**

A 2-phase pilot prospective single-center observational study was designed. During both phases, patients were recruited, and a wearable activity tracker was allocated to gather physical activity data. Patients were categorized into class A (BI≤20; total dependence), class B (20<BI≤60; severe dependence), and class C (BI>60; moderate or mild dependence, or independent). Data preprocessing and machine learning techniques were used to analyze mobility data. A decision tree was used to achieve a robust and interpretable model. To assess the quality of the predictions, several metrics including the mean absolute error, median absolute error, and root mean squared error were considered. Statistical analysis was performed using SPSS and Python for the machine learning modeling.

**Results:**

Overall, 90 patients with complex chronic diseases were included: 50 during phase 1 (class A: n=10; class B: n=20; and class C: n=20) and 40 during phase 2 (class B: n=20 and class C: n=20). Most patients (n=85, 94%) had a caregiver. The mean value of the BI was 58.31 (SD 24.5). Concerning mobility aids, 60% (n=52) of patients required no aids, whereas the others required walkers (n=18, 20%), wheelchairs (n=15, 17%), canes (n=4, 7%), and crutches (n=1, 1%). Regarding clinical complexity, 85% (n=76) met patient with polypathology criteria with a mean of 2.7 (SD 1.25) categories, 69% (n=61) met the frailty criteria, and 21% (n=19) met the patients with complex chronic diseases criteria. The most characteristic symptoms were dyspnea (n=73, 82%), chronic pain (n=63, 70%), asthenia (n=62, 68%), and anxiety (n=41, 46%). Polypharmacy was presented in 87% (n=78) of patients. The most important variables for predicting the BI were identified as the maximum step count during evening and morning periods and the absence of a mobility device. The model exhibited consistency in the median prediction error with a median absolute error close to 5 in the training, validation, and production-like test sets. The model accuracy for identifying the BI class was 91%, 88%, and 90% in the training, validation, and test sets, respectively.

**Conclusions:**

Using commercially available mobility recording devices makes it possible to identify different mobility patterns and relate them to functional capacity in patients with polypathology according to the BI without using clinical parameters.

## Introduction

The Spanish strategy for the approach to chronicity in the National Health System defines patients with complex chronic diseases as patients with 1 or more chronic diseases that present greater complexity in their management due to changing needs that force continuous evaluations and make necessary the coordinated use of various care levels and, in some cases, health and social [1]. Social changes and health advances mean that we are living longer and better and that most diseases affecting us are becoming chronic. Several of them are accumulating, which causes the growing phenomenon of people living with polypathology or complex chronic diseases. This concept includes not only people with the primary disease that triggers other secondary conditions but also those people where 2 or more chronic diseases coexist. It is a population characterized by frailty, polymedication, old age, hyperfrequent use of emergency services, and frequent re-entering. It is estimated that 70% to 95% of the older people in our environment have 1.2 to 4.2 chronic diseases, which constitute the leading death cause in the world (60% of the total) [2]. These patients generate a greater demand for attention in different care settings and use a more significant number of health and social resources. It is predominantly seen in older patients presenting with limiting and progressive diseases (eg, renal or cardiac insufficiency), polypharmacy, and some degree of functional impairment [3].

Functional impairment is one of the most decisive prognostic factors in patients with complex chronic diseases. A more significant functional impairment indicates that the disease is progressing, which requires implementing diagnostic and therapeutic actions that stop the exacerbation of the disease. The functional assessment of patients with complex chronic diseases can be performed using tools such as the Barthel Index (BI) [4], mobility tests, the 4-meter gait test [5], the balance test [6], and the timed “up and go” test [7].

The BI has excellent predictive value for variables such as mortality, hospital admission, and stay length in rehabilitation departments. In addition, it is an indicator to assess the functional and prognostic capacities of patients with complex chronic diseases [8,9]. The BI is a simple measure developed on empirical bases in obtaining and interpreting it. It is about assigning, to each patient, a score based on their degree of dependence to perform a series of basic activities related mainly to the individual’s mobility (eg, moving between the chair and the bed, moving, going up and down stairs, or showering). The total score can vary between 0 (fully dependent) and 100 points (completely independent) [10].

Concerning functional capacity, physical inactivity is defined as the spectrum of any decrease in body movement that reduces energy expenditure toward the baseline level. Physical inactivity affects many aspects of a person, such as respiratory capacity, bones, or the central nervous system, among others, and can even lead to various diseases [11]. In addition, physical inactivity itself decreases the physical fitness of the person, the duration of good health, and the age of onset of his or her first chronic illness. Relative to this, there are several parameters to assess the physical inactivity of the person, such as the number of daily steps, the time spent sitting, or the immobilization of the limbs, among others.

On the other hand, the possible causal relationship between sedentary behavior and mortality due to various causes has been studied. Various studies used accelerometers on the thigh to control the body’s position, and the chances of experiencing illnesses increased for every additional hour of sitting. Regarding limb immobilization in older people, one of the main concerns is the inability to recover the loss of bone strength and muscle mass [12].

Recent technological advances allow mobility monitoring through smartphones or wrist devices, which are widely distributed throughout the population. These devices provide information on the paths, the number of steps, the speed of the march, and the periods of falls, among others. Specific initiatives have tried to apply this information to the health sector. For instance, a multiagent system equipped with sensors has been developed to collect vital signs from patients. This system is intended to facilitate various tasks within the residences of older or disabled people [13]. Additionally, mobility monitoring by sensors in different rooms of the house has been considered to study translations between rooms and measure the length of stay in each room for older patients living alone [14].

Furthermore, individual physical activity can be monitored using accelerometers placed on the patient’s trunk and thigh [15]. At the same time, smartwatches have been used to evaluate movement and gait patterns in patients with Parkinson disease and essential tremor [16]. These advancements are driving the development of more hardware devices to enhance health care delivery and turn the concept of “a doctor in your pocket” into a reality for patients.

We would like to emphasize that using sensors to obtain health information currently has a specific trajectory [17-19]. Mobility has long presented prominent importance when dealing with diseases whose onset and symptomatic progression affect the functional capacity of the subject [20]. Recently, machine learning (ML) techniques are increasingly being considered to characterize the movement, or some particularities of the movement, which can provide relevant information about the patient’s clinical status [21,22]. Some works investigate the relationships between movement and specific clinical pathologies [23,24].

Despite these initiatives, the evaluation of the mobility of patients with complex chronic diseases and their relationship with the functional capacity measured by the BI has yet to be explored [25]. For all these reasons, and with this background, this study aims to develop and validate mobility patterns based on artificial intelligence and the internet of things (IoT) environment, aiming to predict changes in the clinical condition of patients with complex chronic diseases through the prediction of the BI to know the clinical and functional status of the patients.

## Methods

### Study Design and Recruitment

This 2-phase pilot observational study has been designed to analyze how mobility deterioration can reflect changes in the patient’s clinical condition and possible degeneration in the integrated care of patients with complex chronic diseases. To this end, a prospective, single-center, descriptive study was carried out.

Eligible patients met the criteria of chronic patients with complex health needs defined according to the Integrated Patient Care Process of the Andalusian Ministry of Health [26]. Concretely, the study population included patients older than 65 years of age with multimorbidity (ie, diagnosed with at least 2 chronic diseases), and the recruitment took place at the Virgen del Rocio University Hospital of Seville, Spain. In addition, those patients in a situation of agony or those whose vital prognosis was limited, patients with psychiatric disease, and patients or caregivers unable to use mobility devices were excluded from the study. The study subjects were patients of the Internal Medicine Department of the Virgen del Rocio University Hospital of Seville, as part of the Andalusian Health Service, Spain.

**Figure 1:**
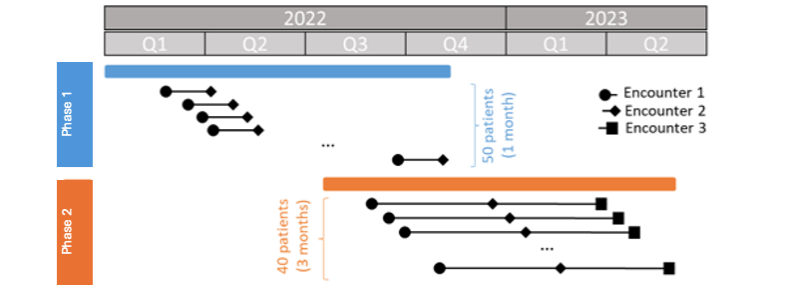
Gantt chart illustrating the temporal progression of the enrollment in the 2-phase clinical study. Each horizontal bar represents the beginning of the patient monitoring and the 1-month or 3-months span, depending on if it is phase 1 or phase 2, before the following BI measurements. BI: Barthel Index; Q: quarter.

The research was conducted in 2 phases. In the initial phase (January to November 2022), a cohort of 50 patients was enrolled, and their BI was measured before the allocation of the wearable activity trackers (WATs), during routine doctor appointments after providing informed consent. Approximately 1 month after the first assessment (encounter 1), the BI was measured again (encounter 2) to evaluate any changes in their functional status (Figure 1). The recruitment was conducted according to different degrees of patients with complex chronic diseases dependence based on the BI measured during the first assessment. In particular, the enrolled patient’s group was classified into 3 groups based on their BI scores: class A included patients with BI≤20 (total dependence), class B comprised patients with 20<BI≤60 (severe dependence), and class C consisted of patients with BI>60 (moderate or mild dependence, or independent) [27].

In the second phase (July 2022 to May 2023), 40 patients were recruited. Similar to phase 1, patients were recruited during doctor appointments, and their BI was measured 3 months after encounter 1 and encounter 2. Therefore, for phase 2 patients, there was an additional encounter 3.

**Figure 2:**
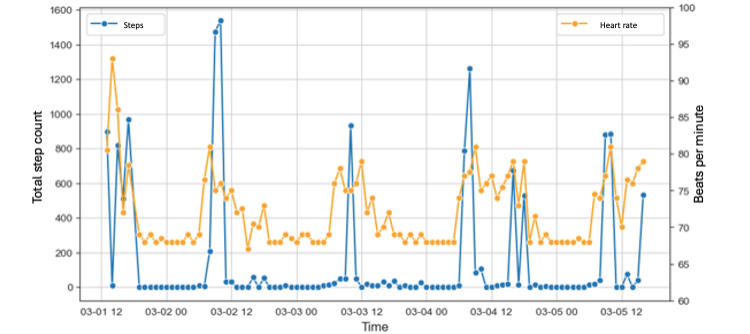
Illustration of data captured by the wearable activity trackers. The line charts show a specific patient’s step count and heart rate daily time series.

An IoT framework was deployed to gather patient mobility data after analyzing the existing devices and applications in the market. The IoT-based infrastructure consisted of using mobile devices and WAT to measure the mobility activities of patients, considering the no or minimal invasion in the development of the daily tasks for the patients under study. The WAT used in this study recorded the step count, the cardiac activity, and the sleep duration from which both the step count and the heart rate were analyzed (Figure 2).

**Figure 3:**
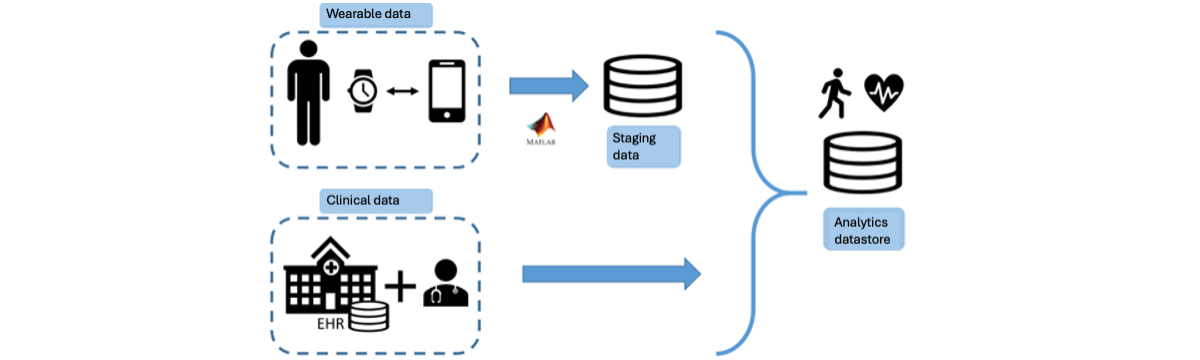
Visual representation of the extract, transform, and load (ETL) process. Incorporating WAT-derived measurements, clinical records from health care systems, and patient-specific data into the analytics datastore used for the model training. EHR: electronic health record; WAT: wearable activity tracker.

Once the 90 patients were included in the study, they were assigned a WAT, and different mobility and functional status tests were conducted. An information system for data storage (Analytics Datastore) was developed. This database allowed both the dumping of the information collected through the WAT and the storage of the relevant clinical information of the patients extracted from the electronic health records (Figure 3). For this purpose, confidentiality protocols of information and the security of the center’s systems were followed and in compliance with the ethical approval obtained by the hospital’s ethics committee.

The study patients’ exposure and clinical variables of interest were analyzed to characterize patient groups regarding mobility, using mobility measurement devices and clinical conditions. Demographic and clinical variables such as diseases, fragility, and polypathology criteria; pharmacological variables; and functional tests such as the BI, balance test, and timed “up and go” test were collected. Statistical analysis was performed using SPSS Statistics software (version 25; IBM Corp) and Python (version 3.10.9; Python Software Foundation) for the ML modeling.

### Data Preparation for the ML Model

The WAT automatically gathered continuous and noninvasive data on a range of parameters, encompassing heart rates, step counts, and sleep duration. However, these raw data must be processed to apply ML techniques. Furthermore, given the potential influence of the walking aids on mobility patterns, patients were classified into 3 distinct groups. The first group encompassed patients using wheelchairs; the second comprised individuals using canes, walkers, or receiving aid from a caregiver during ambulation; and the third consisted of those with no reliance on assistance.

To ensure high data quality, instances where the median heart rate is missing are identified as null, along with the corresponding count of steps. The steps taken within 1-hour intervals are aggregated, and the median heart rate for these hourly intervals is computed. The resulting time series data of hourly step counts are then smoothed by applying a centered rolling window with a window size of 3. Subsequently, the data are grouped by the specific hour, resulting in an average representation of each patient’s activity throughout a 24-hour period. The data used to generate the mean activity profile consist of the information recorded during the 30-day period before encounter 2 requiring at least 14 days’ worth of data to consider the patient in the data set. This approach aims to develop a methodology that allows the estimation of the BI at any given moment using the information collected by the WAT over the last 30 days. Therefore, it holds the potential to provide a more dynamic and real-time assessment of the BI based on continuous monitoring through WAT. Additionally, mobility profiles from encounter 3 served as a production-like test set and were excluded from the model’s training. This strategy evaluated the model’s real-world performance and generalization on unseen data.

The 24-hour mean activity profiles were partitioned into 4-time segments: morning (7 AM-1 PM), afternoon (2 PM-7 PM), evening (8 PM-11 PM), and overnight (midnight-6 AM). This methodology aimed to reduce the dimensionality of the data inputted into the model. Various approaches were considered to reduce dimensionality, including summing the steps within each interval, calculating the mean, and determining the maximum value. The 4-time segments were selected based on the findings of Polo-Molina et al [25], where it was demonstrated that mobility patterns can be categorized into distinct clusters. The study highlighted that the maximum value of steps within each interval aligns with the suggested division, regardless of individual variations in the mobility patterns.

Once the data set was generated, it was divided into training and validation sets, with 70% (n=63) of the records allocated for training and 30% (n=27) for validation. To ensure that the proportions of each group of walking aids were maintained at this ratio, the division was performed within each group, and then the data were combined to create the final training and validation data sets. This approach aimed to ensure representative and well-balanced distributions of walking aids in both sets, allowing for robust evaluation of the model’s performance across different modes of mobility.

### Explainable ML Model

A decision tree regressor has been considered to predict the BI. Decision trees iteratively select variables to maximize information gain or minimize impurity at each decision node, creating a hierarchical structure. Therefore, starting from the whole set of variables, at each split, the training algorithm selects the variable that generates the best split [28].

Moreover, to optimize the performance of the regression model, the hyperparameters were fine-tuned using a cross-validation approach with 7 folds. This technique ensures robustness and selects the optimal settings that yield the best predictive accuracy for the BI. The cross-validation optimization considered the hyperparameters “min_impurity_decrease” (ranging from 0.0 to 1.0 in increments of 0.01), “min_samples_leaf” (from 1 to 10 in steps of 1), and “min_samples_split” (spanning 1 to 10 with an interval of 1).

In addition, to assess the quality of the predictions, several metrics were considered, including the mean absolute error, median absolute error (MAD), and root mean squared error.

Finally, the permutation importance from explanatory variables was computed by permuting individual feature values while measuring the subsequent decline in model performance [28]. This iterative process assigned a score to each feature based on the decrease in predictive power caused by permutations, with elevated scores indicating significant contributions to accurate predictions.

### Ethical Considerations

First, ethical approval was obtained in the health organization based on the regional regulations before involving it in the study execution. Likewise, informed consent procedures were defined, including informed consent and information sheets for the patients who were included in the study. Before starting this study, and based on the ethical and legal regulations, ethical approval was requested from the Ethics Committee of the Virgen del Rocio University Hospital of Seville, Spain. The study protocol, informed consent documents, and information sheets were submitted, and approval from the ethics committee was received. The study began, and patients who met the inclusion criteria were invited to participate after explaining the study procedures. Those who accepted and signed the informed consent and information sheets were included in the clinical study.

In addition, to ensure the protection of the privacy and confidentiality of the study participants, sensible data were anonymized and deidentified. Likewise, confidentiality protocols of information and the security of the center’s systems were followed and in compliance with the ethical approval obtained by the hospital’s ethics committee.

## Results

### Statistical Analysis

A total of 90 patients were included in the study and were classified into 3 categories according to their BI. Concretely, 50 patients were enrolled in the first phase (10 in the BI class A, 20 in BI class B, and 20 in BI class C), and in the second phase, 40 patients were included (20 patients in BI class B and 20 patients in BI class C).

Of the patients, 94% (n=84) had a caregiver, of which 40% (n=34) had a son or a daughter, 32% (n=27) had a spouse, 17% (n=14) had other relatives, and 11% (n=9) had a professional caregiver. The mean value of functional capacity measured by the BI was 58.31 (SD 24.5). Concerning mobility aids, 58% (n=52) of patients did not require it, 20% (n=18) required a walker, 17% (n=15) a wheelchair, 4% (n=4) required a cane, and 1% (n=1) required crutches. The clinical complexity was high with 76 (85%) patients meeting the criteria for patients with polypathology, with a mean of 2.7 (SD 1.25), and 19 (21%) patients met the criteria for patients with complex chronic diseases. A total of 61 (69%) patients met the frailty criteria.

The most characteristic symptoms of this population were dyspnea (n=73, 82%), with 47% (n=42) of patients requiring home oxygen therapy; chronic pain (n=63, 70%); asthenia (n=61, 68%); and anxiety (n=41, 46%; Table 1). The mean number of drugs taken chronically was 12.19 (SD 11.88), with 87% (n=78) meeting the polypharmacy criteria and 70% (n=63) meeting the extreme polypharmacy criteria. Psychotropic drugs were the most consumed pharmacological group (n=29, 33%). Five patients died during the study.

**Table 1:** Description of the baseline characteristics of the study in line with the mobility classification.

Characteristics			Mobility classification															
			BI^a^ ≤20 (total dependence)		20<BI≤60 (severe dependence)							BI>60 (moderate or mild dependence, or independent)						
					Phase 1 (n=20)	Phase 2 (n=20)		*P* value			Phase 1 (n=20)			Phase 2 (n=20)			*P* value	
Age (years), mean (SD)			72.56 (14.5)		79.6 (8)	75.35 (8.9)		.49			78.65 (6.5)			75.2 (6.8)			.90	
Gender (women), n (%)			8 (80)		11 (55)	9 (45)		.40			8 (40)			7 (35)			.74	
Caregiver, n (%)			10 (100)		20 (100)	20 (100)		.12			16 (80)			19 (95)			.15	
BI, mean (SD)			13.89 (4.8)		42.25 (10.12)	50.25 (10.44)		.89			80 (12.1)			80.75 (11.2)			.54	
Balance test, mean (SD)			0 (0)		1.4 (2.1)	1.6 (1.46)		.74			4.6 (1.39)			4 (1.65)			.09	
**30-second chair stand and go, n (%)**									>.99									>.99
	0 points	10 (100)		16 (80)		16 (80)				16 (80)			16 (80)					
	1 point	0 (0)		4 (20)		4 (20)				4 (20)			4 (20)					
**Timed “up and go” test, n (%)**									.47									.06
	No frailty	0 (0)		0 (0)		1 (5)				15 (75)			8 (40)					
	HRF^b^	8 (80)		11 (55)		10 (50)				0 (0)			1 (5)					
	Frailty	2 (20)		9 (45)		9 (45)				5 (25)			11 (55)					
Frailty criteria, n (%)			7 (70)		14 (70)	16 (80)		.46			12 (60)			13 (65)			.74	
PP^c^ criteria, n (%)			10 (100)		19 (95)	18 (90)		.32			14 (70)			16 (80)			.64	
Patient with polypathology categories, mean (SD)			3.3 (1)		2.89 (1.15)	3 (1.48)		.23			2.3 (1.08)			2.35 (1.22)			.98	
PCCD^d^, n (%)			4 (40)		4 (20)	2 (10)		.08			17 (85)			17 (85)			>.99	
Dyspnea, n (%)			5 (50)		17 (85)	18 (90)		.78			18 (90)			17 (85)			.33	
Home oxygen, n (%)			4 (40)		7 (35)	7 (35)		.45			9 (45)			12 (60)			.26	
Chronic pain, n (%)			8 (80)		9 (45)	3 (15)		.001			11 (55)			7 (35)			.2	
Pressure ulcer, n (%)			6 (60)		2 (10)	1 (5)		.54			0 (0)			2 (10)			.15	
Insomnia, n (%)			5 (50)		10 (50)	7 (35)		.17			10 (50)			8 (40)			.37	
Anxiety, n (%)			8 (80)		9 (45)	10 (50)		.66			7 (35)			7 (35)			<.99	
Asthenia, n (%)			8 (80)		14 (70)	15 (75)		.49			12 (60)			12 (60)			<.99	
Anorexia, n (%)			40 (40)		8 (40)	6 (30)		.21			3 (15)			5 (25)			.12	
Nausea and vomiting, n (%)			3 (30)		5 (25)	2 (10)		.12			2 (10)			1 (5)			.29	
Diarrhea, n (%)			4 (40)		2 (10)	1 (5)		.23			1 (5)			1 (5)			<.99	
Number of drugs, mean (SD)			13.11 (3.6)		11.85 (3.6)	12.5 (3.2)		.86			11.8 (3.3)			12.2 (3.6)			.44	
Polypharmacy, n (%)			10 (100)		20 (100)	20 (100)		<.99			19 (95)			19 (95)			<.99	
Extreme polypharmacy, n (%)			8 (80)		15 (75)	16 (80)		.46			15 (75)			16 (80)			.43	
Death, n (%)			2 (20)		2 (10)	0 (0)		.14			1 (5)			0 (0)			.31	

^a^BI: Barthel Index.

^b^HRF: high risk of fall.

^c^PP: polypathological patient.

^d^PCCDs: patients with complex chronic diseases.

Regarding the baseline characteristics, comparing the different phases 1 and 2 categories, significant differences were only found in the presence of pain in the classification BI class B (Table 1). In that category, the mean BI at the beginning of the study was 50.25 (SD 10.44), with an increase at the end of the study to 63.53 (SD 28.92). In BI class C, an initial BI of 80.75 (SD 11.27) and a final BI of 86.76 (SD 15.806) were observed. In the initial balance test for BI class B, the value was 1.6 (SD 1.46) points and 4.8 (SD 1.61) points at the end of the study, while in the BI class C, the initial value was 4 (SD 1.6) and the final value was 5.5 (SD 2.1).

### Data for the ML Model

Following the aforementioned methodology, the patient cohort was reduced to 54 patients, taking into account only those who had at least 14 days’ worth of records before the doctor’s appointment. The principal factors contributing to this lack of data were predominantly attributed to mortality or patients becoming bedridden, subsequently ceasing to use the wristband. Mobility profiles corresponding to encounter 3 were used as a production-like test in a cohort of 21 patients. The best model results for defining the average 24-hour activity profile were obtained using the maximum value of steps in each interval and the type of walking aid if needed. Table 2 presents the complete set of variables used for training the model. The mean BI in the training and validation sets are 66.5 (SD 23.4) and 65.0 (SD 24.1), respectively. In contrast, the mean value in the test set is notably higher at 85.0 (SD 22.5).

**Table 2:** Description of the candidate features used to train the regression tree model.

Variable name	Description
morning_max	Maximum number of steps recorded during the morning period
afternoon_max	Maximum number of steps recorded during the afternoon period
evening_max	Maximum number of steps recorded during the evening period
overnight_max	Maximum number of steps recorded during the overnight period
no_walking_aid	Avoidance of any type of walking aid
cane_or_walker	Use of either a cane, walker, or caregiver’s help for walking
wheelchair	Use of wheelchair

**Figure 4:**
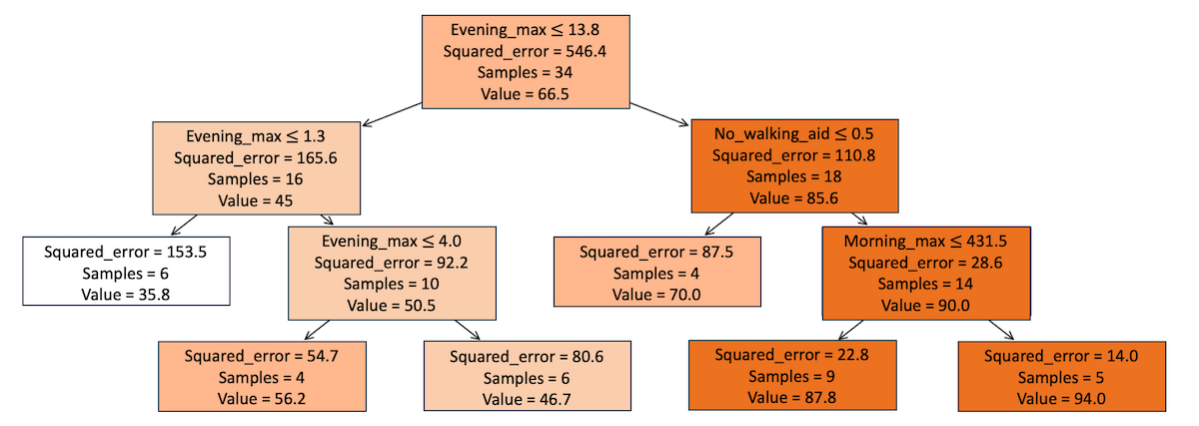
Regression tree model visualization detailing the decision paths within the fitted model. Nodes represent decision points, indicating the sample sizes at each node and the square error committed if the value considered is as the prediction.

**Figure 5:**
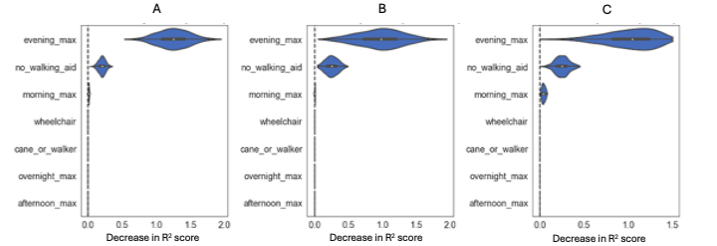
Descending variable importance ranking for the fitted model. Each violin plot represents the distribution of the decrease in R2 score when a single feature value, represented in the y-axis, is randomly shuffled. The importances of the permutations are labeled A (Train set), B (Validation set) and C (Test set).

The fitted model, whose parameters were selected through cross-validation, is a decision tree regressor with a depth of 3, minimum impurity decrease of 0.0, minimum samples in a leaf node of 2 and minimum samples in a split of 9 (Figure 4). Among the features considered, the most important variables for predicting the BI were identified as the maximum step count during the evening and morning periods, and the absence of a mobility device. These key predictors were determined based on their significant impact on the functional status of the patients (Figure 5 [28]).

**Figure 6:**
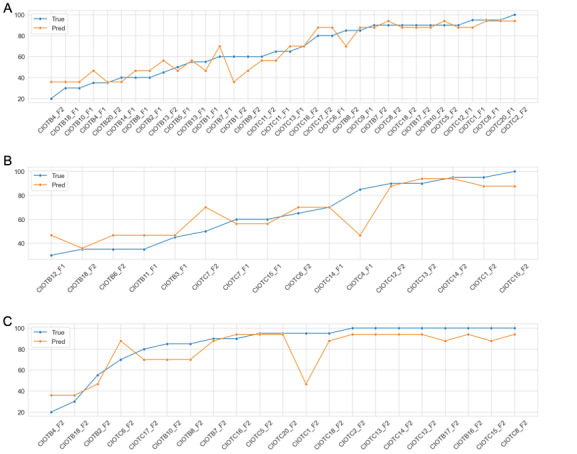
Comparative of real versus predicted values for training (A), validation (B), and test (C) ordered by increasing Barthel Index values. The horizontal axis of the graphs represent the values for the different subjects of the study. To de-identify them, CIOT (Chronic patient intenet of things) project identifiers were used, followed by the B (patients with Barthel Index >20 and ≤ 60 [severe dependence]) or C (patients with Barthel Index >60 [moderate or mild dependence, or independent]) codes with the number assigned to each subject.

Based on the results in Table 3, the model exhibits consistency in MAD with a value close to 5 in the training, validation, and test sets. Furthermore, according to Figure 6, when observing the predicted values compared to the real ones, the model does not present a significant difference between the predicted and the real BI.

**Figure 7:**
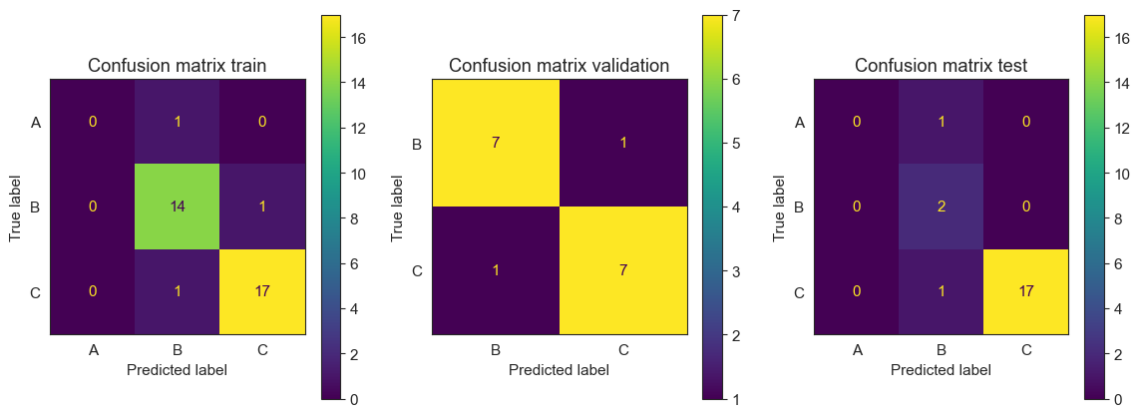
Multiclass confusion matrix for training, validation, and test, respectively. Each matrix showcases the count of accurately predicted patients, contrasting with those whose predicted labels differ from the true labels.

Once the BI prediction was performed, the intervals defining each BI class were further considered. Subsequently, a classification prediction is carried out by converting the predicted value into its corresponding class label, thereby assigning the appropriate class to the given BI prediction. As observed in Table 3 and Figure 7, in the training set, the model achieved precision, recall, and *F*_1_-scores of 0.88, 0.93, and 0.90 for class B, respectively. For class C, the model obtained precision, recall, and *F*_1_-scores of 0.94 for all 3 measures. However, for class A, all the metrics were 0.00 due to the limited support for that class (only 1 instance). In the validation set, the model demonstrated consistent performance with precision, recall, and *F*_1_-scores of 0.88 for both class B and class C. On the other hand, in the data coming from the test set, the model achieved precision, recall, and *F*_1_-scores of 0.5, 1, and 0.67 for class B, respectively. For class C, the model obtained precision, recall, and *F_1_*-scores of 1, 0.94, and 0.97, respectively. Finally, only 1 member from class A was predicted as class B.

**Table 3:** Training, validation, and test errors and accuracy of the model when transforming the predicted values to class A, B, and C.

	Train	Validation	Test
Accuracy	91%	88%	90%
MAE^a^	6.30	8.75	10.10
MAD^b^	5.42	4.50	6.00
RMSE^c^	8.13	12.99	14.05

^a^MAE: mean absolute error.

^b^MAD: median absolute error.

^c^RMSE: root mean squared error.

## Discussion

The use of mobility recording devices identifies different mobility patterns and relates them to functional capacity in patients with polypathology. One of this study’s findings is improving functional capacity measured by the BI in patients after using the mobility devices for 6 months in a real environment. This improvement is slight, 13 points in the case of moderate dependence and 6 points in mild dependence. We believe that it reflects an effect of the empowerment experienced by patients with the use of mobility devices. Therefore, the use of such mobility monitoring devices may have a potential impact on the management of complex chronic patients and could be included as part of clinical follow-up practices. Specifically, during the study execution in a real environment, patient empowerment was related to patient participation in decision-making, gaining control, and learning about their health.

In addition, patients’ sense of empowerment was related to less frustration with the technology [29]. This effect is well-known in the literature. A systematic review of 71 articles analyzing patients’ expectations of digital tools found that mobile apps increase patient engagement and motivation, especially when they can visualize parameters graphically and thus monitor their outcomes over time [30]. The evidence is scarce in patients with polypathology; but in another study of our group, we found similar results with slight improvements in functional capacity [31]. This empowerment is going to help patients in the self-management of their diseases. Health status has shown that incorporating digital technology into patients’ lives increases their awareness of lifestyle behaviors, which has helped them understand how to manage their health better and promote autonomy [32]. Longer term studies are needed to confirm this benefit, although it could be an alternative to integrate into the clinical practice of these patients to minimize their functional impairment.

Another possible beneficial effect of continuous monitoring of the functional capacity of patients with complex chronic diseases is the early detection of functional deterioration that may be the beginning of exacerbations of their diseases. If these data are integrated into the health care computer system, alarm situations could be determined that would allow early reaction by health staff to treat such exacerbation, prevent its progression, and minimize the functional deterioration that could be caused to the patient.

It should be noted that commercially available mobility monitoring devices have been used for this study and devices specifically designed for the study were not required. This favors cost reduction when considering the implementation of activity monitoring in patients with polypathology in real-world settings. Since the population with mobility devices is growing, with 515 million units sold in 2022, and patients with polypathology are a population that continue to increase and probably have their own mobility measurement device [33], the costs are thus reduced by integrating data in the informatics systems of the different health care organizations.

Another contribution of this study was to determine that mobility devices do not accurately recognize patients’ steps when using walking aids. For that reason, and to avoid this possible bias, patients were categorized into 3 groups depending on the walking aids. Additionally, caregivers assisting patients during physical activity have been classified similarly to canes or walkers due to their similarity in providing walking support. After the inclusion of an extra variable with the group to which the patient belongs, the ML model has managed to alleviate these limitations, achieving a good performance.

Concerning the data for the ML model, the use of the maximum value of steps taken in each of the 4 intervals defined (morning-afternoon-evening-overnight) yielded the most promising outcomes. This finding can be attributed to the limited and typically short-lived movements observed in patients with complex chronic conditions, which rarely extend beyond an hour. By leveraging the maximum step count within each time segment, we effectively capture the most significant and representative activity level during that period, thus optimizing the model’s performance. The selected intervals concur with the typical Spanish timetable for meals. Furthermore, the mean and SD data values found in the training and validation sets were similar, suggesting consistent levels of variability in both data sets. Moreover, including the heart rate information, measured by the WAT, and its relationship with the step count is proposed as a future study. Therefore, it could help to distinguish the requirement of the physical activity considering the cross-correlation or the cosine similarity between the step count and the heart rate.

Partitioning the data set into 7 equally sized subsets, with each fold serving as a validation set while the remaining folds are used for training, ensures robustness in selecting the optimal settings that yield the best predictive accuracy for the BI [34]. The hyperparameters play a crucial role in controlling the complexity and generalization of the decision tree model. By tuning these hyperparameters, the cross-validation process aims to find the optimal combination that balances model complexity and performance, resulting in a decision tree model with improved predictive capabilities [28].

Using a decision tree as a regression model holds paramount importance in the biomedicine field, particularly due to the necessity of using a highly interpretable model that can be effectively used and comprehended by the medical team [35,36]. The interpretability of the model enables medical professionals to understand the underlying decision-making process and gain insights into the factors influencing the predictions. This transparency fosters trust and facilitates collaboration between the model and the clinicians. Although more complex approaches exist, such as random forest or extreme gradient boosting, the ability to provide better results than decision trees in terms of accuracy most of the time, their lack of interpretability, and the limited sample size of this study advise against its use. Under these circumstances, a valid alternative to regression trees is multiple linear regression. However, a linear regression model based on the same variables as the decision tree has been performed, yielded inferior results.

Regarding the model performance, upon comparing the results obtained from the model’s predictions with the ground truth, the decision tree model generates accurate predictions. Therefore, the decision tree model can assess the functional capacity of patients based on data collected from the WAT. As observed, the errors remain similar among the training, validation, and test sets. Hence, this confirms that the model can generalize to unseen cases.

There is an imbalanced distribution of classes in the production-like test set, as shown in Figure 7. This discrepancy arises from the natural transition of patients between classes B to C and A, due to changes in the BI, coupled with the criterion of minimum data required for data set inclusion. It is noteworthy that the test set could have been randomized to achieve an even distribution of class numbers, akin to the training and validation sets. However, given the primary goal of evaluating the model’s performance in a realistic production setting, this randomization was deliberately omitted.

In addition, it is worth noting that the MAD is the most suitable performance measure in this case. This choice is justified by the variability in step measurements captured by the WAT and the relatively small data set. It is possible that a patient with inaccurately measured data could significantly influence the error measure, particularly in terms of absolute or squared errors. By considering the MAD as the primary metric, we mitigate the impact of outliers or measurement inconsistencies, ensuring a more robust evaluation of the model’s performance in predicting the BI.

On the other hand, when considering the performance obtained in the classification problem, the accuracy remains consistent in training, validation, and test sets. The main objective of performing regression followed by classification into classes A, B, and C is due to the continuous nature of the BI variable. When aggregated into these 3 intervals, estimating BI solely through classification becomes complex, as small differences in BI values may result in a class label change. Therefore, regression allows the model to capture the underlying continuous relationship within the BI data, enhancing its ability to make more accurate and robust predictions while assigning the appropriate class labels based on the predicted values.

Furthermore, the model does not need to include clinical information such as specific disease, number of comorbidities, severity of disease, and so forth. Therefore, it can be regarded as a general model for patients with complex chronic diseases without specific clinical data, facilitating the development of a methodology that allows estimating the BI at any given moment using the information collected by the WAT over the last 30 days.

This study has several limitations. Since this was a pilot study with a small number of patients, the results should be confirmed by studies with a larger population. Prospective studies are needed to analyze whether identifying mobility changes and their transfer to health care systems can have care implications and improve the health status of patients with multiple pathologies. A notable element is that the bracelet does not register well the physical activity of patients who use a cane or crutches (or a wheelchair) since it cannot measure steps. A priori, this could be a limitation of the study. Still, adjusting the model by identifying walking aid devices and evaluating other parameters makes it possible to identify and predict mobility patterns in these patients.

In conclusion, using commercially available WATs makes it possible to identify different mobility patterns and relate them to functional capacity in patients with polypathology according to the BI without using clinical parameters.

## Abbreviations

BI: Barthel Index
IoT: internet of things
MAD: median absolute error
ML: machine learning
WAT: wearable activity tracker

## Data Availability

The data sets generated during and analyzed during this study are not publicly available because there is no Ethics Committee approval for this purpose, since the received Ethics Committee’s approval was related to carrying out this study. However, they could be available from the corresponding author on reasonable request and this authorization shall then be requested.
